# MERIT reveals the impact of genomic context on sequencing error rate in ultra-deep applications

**DOI:** 10.1186/s12859-018-2223-1

**Published:** 2018-06-08

**Authors:** Mohammad Hadigol, Hossein Khiabanian

**Affiliations:** 1 Center for Systems and Computational Biology, Rutgers Cancer Institute of New Jersey, Rutgers University, New Brunswick, NJ USA; 2 Department of Pathology and Laboratory Medicine, Rutgers Robert Wood Johnson Medical School, Rutgers University, New Brunswick, NJ USA

**Keywords:** Deep sequencing, Sequencing noise, Genomic context, Polymerase fidelity, Optimal depth

## Abstract

**Background:**

Rapid progress in high-throughput sequencing (HTS) and the development of novel library preparation methods have improved the sensitivity of detecting mutations in heterogeneous samples, specifically in high-depth (> 500×) clinical applications. However, HTS methods are bounded by their technical and theoretical limitations and sequencing errors cannot be completely eliminated. Comprehensive quantification of the background noise can highlight both the efficiency and the limitations of any HTS methodology, and help differentiate true mutations at low abundance from artifacts.

**Results:**

We introduce MERIT (Mutation Error Rate Inference Toolkit), designed for in-depth quantification of erroneous substitutions and small insertions and deletions. MERIT incorporates an all-inclusive variant caller and considers genomic context, including the nucleotides immediately at 5 ^′^and 3 ^′^, thereby establishing error rates for 96 possible substitutions as well as four single-base and 16 double-base indels. We applied MERIT to ultra-deep sequencing data (1,300,000 ×) obtained from the amplification of multiple clinically relevant loci, and showed a significant relationship between error rates and genomic contexts. In addition to observing significant difference between transversion and transition rates, we identified variations of more than 100-fold within each error type at high sequencing depths. For instance, T >G transversions in trinucleotide GTCs occurred 133.5 ± 65.9 more often than those in ATAs. Similarly, C >T transitions in GCGs were observed at 73.8 ± 10.5 higher rate than those in TCTs. We also devised an *in silico* approach to determine the optimal sequencing depth, where errors occur at rates similar to those of expected true mutations. Our analyses showed that increasing sequencing depth might improve sensitivity for detecting some mutations based on their genomic context. For example, T >G rate of error in GTCs did not change when sequenced beyond 10,000 ×; in contrast, T >G rate in TTAs consistently improved even at above 500,000 ×.

**Conclusions:**

Our results demonstrate significant variation in nucleotide misincorporation rates, and suggest that genomic context should be considered for comprehensive profiling of specimen-specific and sequencing artifacts in high-depth assays. This data provide strong evidence against assigning a single allele frequency threshold to call mutations, for it can result in substantial false positive as well as false negative variants, with important clinical consequences.

**Electronic supplementary material:**

The online version of this article (10.1186/s12859-018-2223-1) contains supplementary material, which is available to authorized users.

## Background

The rising utilization of high-throughput sequencing (HTS) in clinical oncology has transformed our understanding of cancer evolution and has provided clinicians with an invaluable tool for precise diagnosis and prognosis.

In clinical cancer genomic testing, target-capture library preparation assays are favored over whole genome or whole exome sequencing approaches because of their lower cost in obtaining higher sequencing depth – the number of reads covering a specific locus [1]. High-depth DNA sequencing enables confident detection of small clones of somatically mutated cells in heterogenous tumor samples, where in addition to genomically diverse cancer cells, contaminating normal cells may also be present. Using polymerase chain reaction (PCR)-based amplicon or hybridization-capture enrichment techniques, clinical-grade cancer sequencing panels are capable of producing 500 to > 10,000 reads mapping to each targeted locus [2, 3]. Specifically, a minimum average depth of 500 × is strongly advised by regulatory bodies for reliable detection of somatic mutations with variant allele frequencies (VAFs) as low as 5% in tumor specimens [4].

The power to detect small clones in heterogenous samples may improve by increasing depth; however, confident detection and differentiation of true mutations with low VAFs, e.g., < 0.1%, from the sequencing artifacts remains a challenge. HTS errors are dominated by misreading a base within the instrument or nucleotide misincorporations during library enrichment with PCR. Differential rate of substitution errors in HTS has been observed and attributed to common DNA damaging events such as spontaneous deamination, presence of oxidized bases in cells in addition to *ex vivo* oxidation during DNA extraction [5], or short-lived high temperatures during acoustic shearing [6]. Such events often lead to higher rates of transitions versus transversions [7–11] or increased number of errors in specific genomic contexts. These differences can be more pronounced at higher sequencing depths and directly impact the sensitivity for detecting true mutations with low VAFs. Here, we hypothesize that the genomic context of substitution errors, i.e., the nucleotides immediately at their 5 ^′^ and 3 ^′^, is a determinant factor in estimating their rates at high sequencing depths. To this end, we generated ultra-deep sequencing data (1,300,000 ×) and developed MERIT (Mutation Error Rate Inference Toolkit), a comprehensive pipeline designed for in-depth quantification of erroneous HTS calls. Using MERIT, we show a significant relationship between substitution error rates and their sequence contexts. In addition to observing more than three orders of magnitude difference between transition and transversion error rates, we identify variations of more than 130-fold within each error type at high sequencing depths. We also propose an *in silico* depth reduction approach to provide insights on estimating optimal depth – where sequencing errors exist at rates similar to those of true mutations. Finally, we propose an assay for detailed assessment of nucleotide-incorporation fidelity for four high-fidelity DNA polymerase molecules.

## Methods

### DNA sample

We obtained HapMap NA19240 human genomic DNA (5 *μ*g) from Coriell, purified from immortalized lymphocytes using the Qiagen Autopure LS instrument in TE buffer (10 mM Tris, pH 8.0/1 mM EDTA) with concentration of 301 ng/L. We assessed sample quality and concentration using Nanodrop and Qubit dsDNA assays before library preparation.

### DNA polymerase enzymes and primer design

We used four high-fidelity DNA polymerase enzymes – NEBNext^®;^ High-Fidelity 2X PCR Master Mix (Hi-Fi 2X), NEBNext^®;^ Ultra^TM^ II Q5^®;^ Master Mix (Ultra II), KAPA HiFi PCR kits with ReadyMix (KAPA), and Invitrogen^TM^ Platinum^TM^ SuperFi^TM^ DNA polymerase (SuperFi) – for PCR amplification. We designed the primers using Primer3 [12] to target four loci in the *TP53* and *SF3B1* genes such that the paired-end reads (R1 and R2) are significantly overlapped (Additional file 1: Tables S1 and S2).

### PCR amplification, indexing, and sequencing

We performed twenty PCR cycles using the Hi-Fi 2X, KAPA, and SuperFi polymerases, and 16 cycles using the Ultra II polymerase in the first round of amplification (Additional file 1: Table S3). The cycle numbers were determined after initial PCR amplification tests in order to obtain similar amount of DNA for each enzyme. The second round of PCR for multiplexing and cluster generation included seven cycles for all four polymerases (Additional file 1: Table S4). After each PCR amplification, AMPure Bead cleanup was performed. First, 0.4 × ratio (20 *μ*L AMPure bead to 50 *μ*L PCR product) was used to remove gDNA and larger fragments (i.e., > 600 bp). For the saved supernatant, additional 80 *μ*L AMPure Bead was added to bring the total to a 2 × ratio. The beads were eluted with 22 *μ*L EB (10 mM Tris, pH 8.0). The annealing temperature of 66°C was determined based on the product specificity and yield for all polymerases after performing gradient PCR optimization at eight different temperatures (Additional file 1: Figure S3). Qubit quantification and Bioanalyzer analysis were performed for quality assessment. Custom amplicon-based sequencing and library preparation were performed at GeneWiz (South Plainfield, NJ) using Illumina HiSeq2500 Rapid Run.

### MERIT: a comprehensive error rate estimator

Comparative performance analysis of the commonly used HTS variant callers [13–15] suggests a significant disagreement between their identified variants [16–18]. These differences are mainly rooted in each pipeline’s specific filtering and statistical methodology. For example, a number of filters is automatically applied to reads by HaplotypeCaller implemented in the Genome Analysis Toolkit (GATK) [13] to exclude uninformative reads from the analysis. This practice is aligned with the goal of the majority of variant callers, which is distinguishing true mutations from the artifacts. However, for a precise quantification of the sequencing noise, i.e., error rate profiling, all the reads need to be included in the analysis as the ultimate goal is understanding the nature of artifacts. SAMtools [14] is the basis of a number of alignment-based variant callers [19, 20], and has high flexibility for changes in its filters. Therefore, MERIT uses SAMtools to identify all positions with alternate alleles from the aligned, indexed sequencing reads. By extracting allele frequencies of substitutions directly from the Pileup file generated by SAMtools mpileup, we make sure all reads are included in the analysis.

As MERIT is designed for ultra-deep HTS applications, the input options of its SAMtools mpileup are set to accommodate high depths while providing the users the ability to modify these parameters based on each sequencing data’s characteristics. Additional file 1: Table S5 summarizes the default input parameters of SAMtools mpileup versus those used in MERIT. These parameters allow MERIT to probe SAMtools Pileup data and extract sequencing information for all substitutions, even when they are present in only a single read amongst tens of thousands. Accurate identification of indels is a challenging problem [21, 22] and beyond the scope of this work. Specifically, SAMtools’s filtering criteria in introducing and extending gaps, could affect calling complex indels, especially insertions, rendering error rate estimates sequencing depth-dependent.

**Fig. 1:**
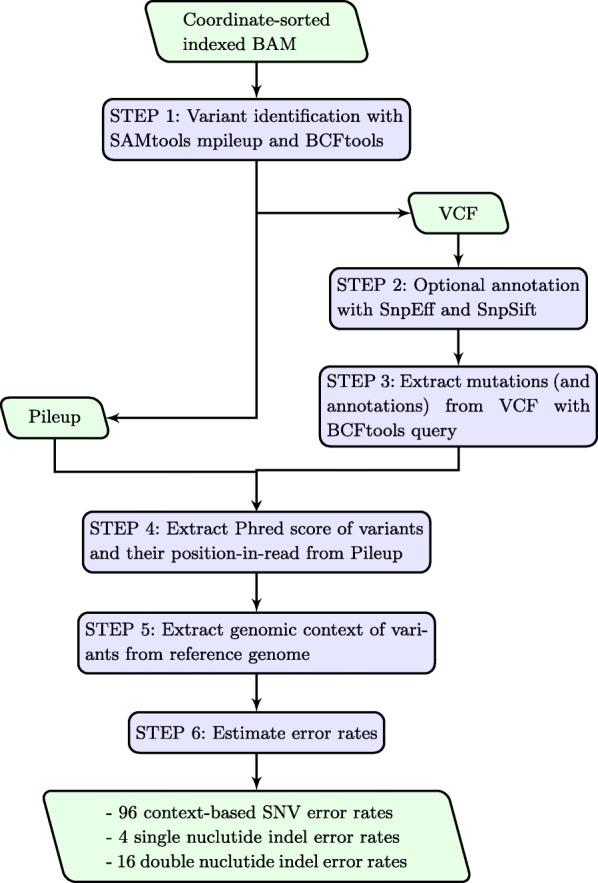
MERIT’s workflow. MERIT is designed for comprehensive characterization of the sequencing error rate in ultra-deep HTS applications

Next, MERIT obtains the Phred quality score of base substitutions as well as the average Phred quality of bases before and after indels. These quantities are not provided in the VCF files generated by SAMtools. Of note, we observed that the alternate allele and total depths at indel loci are only accurate in SAMtools’s Pileup files and not in its VCF. Therefore, to ensure allele frequency accuracy for both indels and substitutions identified, MERIT extracts the reference and alternate alleles’ depths as well as the total depths for all the variants from the Pileup file. MERIT also extracts the position-in-read for all variants. Such information, especially in hybrid-capture sequencing, helps to better quantify the source of errors in HTS platforms. An optional annotation step is also available. Finally, MERIT obtains the genomic context of the variants from the reference genome, including the nucleotides immediately at their 5 ^′^ and 3 ^′^, and estimates error rates for 96 possible single nucleotide substitutions as well as four single-base and 16 double-base insertions/deletions (indels). Details of MERIT’s workflow are shown in Fig. 1.

### Error rate estimation

We used a single HapMap sample to generate ultra-deep data, and although there may be small, uncharacterized variations within initial cell population, we assumed that all detected variants were errors accumulated in library preparation or during sequencing.

We considered context-specific erroneous base calls at each locus to follow a binomial distribution. More precisely, the probability of a single nucleotide *X*_*i*_ with the genomic context *ZX*_*i*_*Z*^′^, *Z*,*Z*^′^∈{*A*,*C*,*T*,*G*}, in a specific locus *i* being misread as *Y*_*i*_, i.e., $P_{ZX_{i}Z' \rightarrow ZY_{i}Z'}$ followed

$$P_{ZX_iZ' \rightarrow ZY_iZ'}=P(x_i|n_i,p)= {{n_i}\choose{x_i}}p^{x_i}(1-p)^{n_i-x_i}, $$

where *p* is the combined PCR and sequencing error rate and *n*_*i*_ and *x*_*i*_ are the total read depth and the number of erroneous calls at position *i*, respectively. Assuming a position-independent *p*, the probability of observing *m* instances of *ZXZ*^′^→*ZYZ*^′^ error within each sample was then given by

1 $$\begin{array}{*{20}l}  P_{ZXZ' \rightarrow ZYZ'} &=P\left(\sum\limits_{i=1}^m x_i|\sum\limits_{i=1}^m n_i,p\right)\\ &= {{\sum_{i=1}^m n_i}\choose{\sum_{i=1}^m x_i}}p^{\sum_{i=1}^m x_i} \left(1-p \right)^{\sum_{i=1}^m \left(n_i- x_{i}\right)}. \end{array} $$

(See Remark 1 in Additional file 1 on the sum of binomial random variables.) For the case of indels, a binomial model was used to describe the error rate as well, but instead of categorizing them based on their context, indels were classified based on the type of inserted/deleted base, as no differential error rates were observed for context-specific indels.

### Polymerase fidelity estimation

The estimated error rate in Eq. (1) has a unit of [error/base]. It is also common to report the fidelity of polymerase enzymes as [error/base/doubling] in the literature where template doubling *d* is given by

$$ 2^d = \frac{\textrm{final DNA amount after PCR}}{\textrm{starting DNA amount for PCR}}. $$

Since precise amounts of input and output DNA were known for our experiment in its second round of PCR, we calculated template doubling and estimated polymerase replication efficiency as the ratio of template doubling *d* over the number of PCR cycles performed (Additional file 1: Table S4). To obtain the total amount of template doubling after performing two rounds of PCR amplification, the total number of PCR cycles were multiplied by the polymerase efficiency which resulted in 20.83, 16.19, 16.87, and 20.98 total template doubling for the Hi-Fi 2X, Ultra II, KAPA, and SuperFi polymerases, respectively.

### Alignment and merging

We cleaned the paired-end (PE) reads of adapters using bcl2fastq Conversion Software (v2.17), and aligned them to the reference human genome hg19 assembly using the Burrows-Wheeler Aligner (BWA) tool [23] (bwa sampe for PE and bwa samse for merged reads along with bwa aln). We then merged the PE reads that properly mapped to the targeted loci. In our merging scheme, if R1 and R2 reads did not match at a base, an N was assigned for that position. We discarded read pairs with smaller than 50 base overlaps or with more than five mismatches. We calculated Phred quality score (*Q*) of a successfully merged locus as the sum of the qualities in R1 and R2 reads since these are independent events; *Q* is given by *Q*=−10 log10*p* where *p* is the probability that the base is called incorrectly. Merged reads were then mapped to the reference human genome hg19 assembly, and were filtered so that they were uniquely mapped (BWA tags X0:1 and X1:0). Finally, in order to make a fair comparison between the error rate of merged and PE reads, we only considered PE reads that were merged successfully and uniquely mapped. Additional file 1: Table S2 represents the average depth of merged and PE reads in different loci. To assess the effect of alternate alignment approaches, we tested Bowtie [24] in addition to BWA to map the merged reads to the reference genome.

### *In silico* depth reduction

The sequencing assay was designed to obtain an average depth of > 1,000,000× bp, but for some amplicons the average depth was substantially larger (Additional file 1: Table S2). Therefore, an *in silico* depth reduction procedure was performed to reduce the high depths and more importantly, generate enough independent samples to estimate low error rates confidently. It should be noted that one of the main hurdles in error rate estimation of high fidelity polymerases via HTS is the lack of signal as errors occur infrequently with increased fidelity, hence, a large number of samples is required to accurately estimate errors. As performing ultra-deep sequencing on a large number of samples is not cost-effective, alternatively, *in silico* data at lower depths can be generated from one ultra-deep sequencing run by randomly selecting reads from the original raw sequencing data.

### Clinical samples

We obtained 29 hematopoietic samples collected from 9 patients with chronic lymphocytic leukemia, previously analyzed by amplicon deep-sequencing (NCBI BioProject PRJNA411889). These samples were sequenced using a custom 88-gene panel, targeting 92 amplicons on Illumina HiSeq (2x150bp) at GeneWiz (South Plainfield, NJ) (Supplementary Table 5 in [25]). The reads were cleaned, merged, and aligned to the reference genome as previously described [25]. We removed previously detected germline and somatic mutations to ensure that the remaining variants represented only the errors.

## Results and discussions

### Impact of merging reads on context-specific error correction

**Fig. 2:**
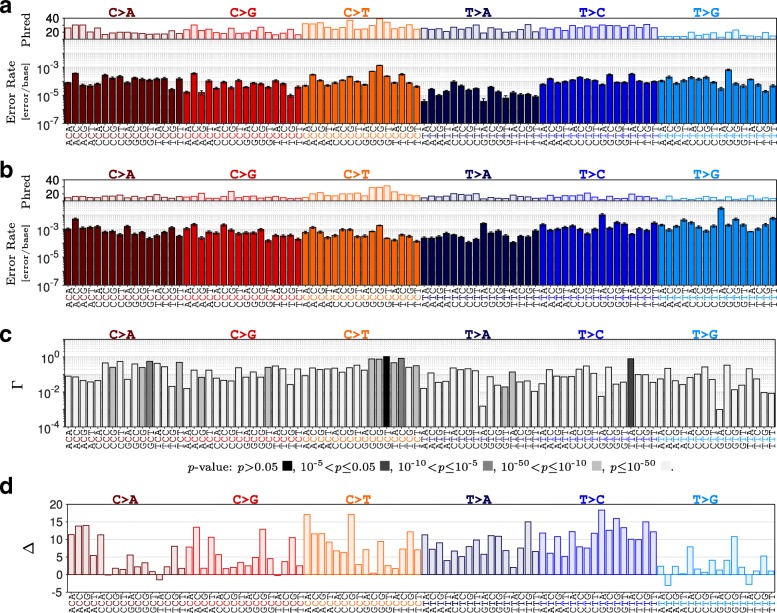
Estimated context-specific substitution error rates for polymerase Hi-Fi 2X. **a**) R1 reads. **b**) R2 read. **c**) *Γ*, the ratio of error rate in R1 over R2. *P*-values were computed by performing a two-tailed z-test. **d**) *Δ*, the difference between their corresponding Phred quality scores. We reduced the depth of paired-end reads to approximately 1,300,000 × through an *in silico* depth reduction procedure. Results were obtained by averaging over 100 independent samples to establish error bars which indicate one standard deviation from the average

**Fig. 3:**
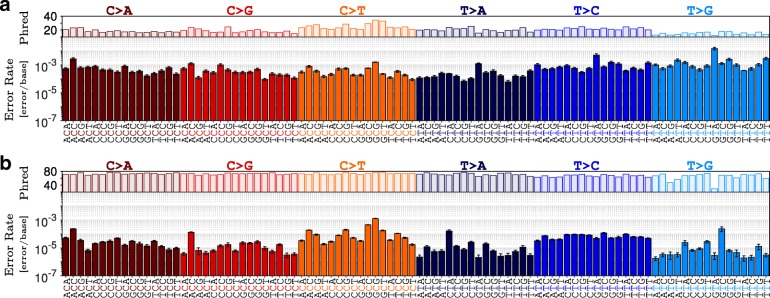
Merging of overlapped PE reads reduces context-specific error rates. **a**) R1 and R2 reads. **b**) Merged reads. Depth of merged reads for polymerase Hi-Fi 2X were reduced *in silico* to approximately 650,000 ×. Error bars indicate one standard deviation from the average of 100 independent sub-samples

Independent analysis of R1 and R2 reads at 1,300,000 × indicated significant variations in estimated error rates across 96 possible sequence contexts (Fig. 2). High error rates and low Phred quality scores observed in R2 relative to R1 may be associated with sequencing errors caused by misreading a base, attributed to image analysis biases [26] or phasing/pre-phasing [11]. These sequencing errors that dominated the R1 and R2 profiles can be distinguished from polymerase errors by merging the overlapped paired-end reads [27–29]. Merging, however, cannot eliminate errors randomly accumulated during the amplification processes and present in both reads. In contrast to higher rates of transversion versus transition errors in paired-end reads (Fig. 3a), the remaining PCR-related errors in the merged reads were dominated by transitions, often with high Phred quality scores (Fig. 3b). MERIT provides further insight for profiling these errors, which are the main hurdle in distinguishing real mutations from sequencing noise:

- Merging R1 and R2 reads lowered all the context-specific error rates. The highest reduction in rate was observed for GTA >GGA transversions (5,025 ± 2,794×) while GCG >GTG transition errors only improved by a factor of 1.22 ± 0.07×. Moreover, these improvements were context-specific. For example, T >A transversion in GTA trinucleotides showed substantial reduction (568 ± 249×) compared to those in CTAs (1.43 ± 0.31×).
- Transition errors occurred at higher rates relative to transversions, in agreement with previous reports [7–11]. This difference was pronounced further when errors were classified based on their context, denoting a rate of 1.29 ± 0.04×10^−3^ [error/base] for GCG >GTG versus that of 2.17 ± 0.92×10^−6^ [error/base] for GTA >GAA (Fig. 3b). MERIT also revealed considerable variation within each substitution type. For example, T >G transversions in GTCs occurred 133.5 ± 65.9× more often than those in ATAs. Similarly, C >T transitions in GCGs were observed at 73.8 ± 10.5× higher rate than those in TCTs (Fig. 3b).
- The rate of C >A errors in ACCs was the highest of all such transversions. These errors are linked to the conversion of guanine to 8-oxoG resulting in mismatched pairing with adenine [30, 31]. Oxidation of guanine to 8-oxoG happens naturally in living cells and can be increased by DNA damaging factors such as acoustic shearing [32].
- Merging R1 and R2 can correct for the low quality erroneous bases associated with sequencing errors. Our analysis suggests that such sequencing errors can be identified and eliminated based on their quality, when merging the reads is not possible (e.g., in hybrid-capture-based sequencing where read pairs are not designed to necessarily overlap).

Finally, we tested whether an alternative alignment method, such as Bowtie [24], would affect error rate estimations, and found minimal changes across the 96 genomic contexts (Additional file 1: Figure S4).

### Effect of mutation context on amino acid variations

**Fig. 4:**
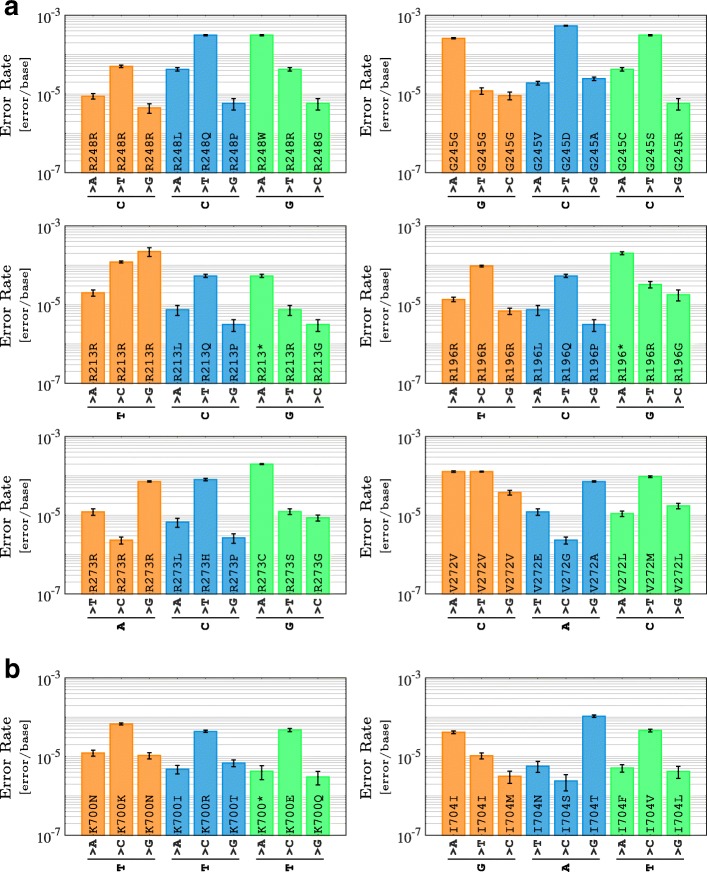
Significant variation in error rates for possible amino acid changes at individual codons. **a**) Six frequently mutated residues in the *TP53* gene. **b**) Two hotspot residues in the *SF3B1* gene. The higher the rate of error for a specific base change, the lower the power to distinguish true mutations from sequencing artifacts at its position. Here, the error rates represent the amplification by the Hi-Fi 2X polymerase. Error bars represent one standard deviation from the mean of 100 independent sub-samples

In a single codon, the context-specific rate of error for each base change directly affects the sensitivity of detecting the resulting amino acid variation. Our data indicated that the most commonly mutated residues in *TP53* and *SF3B1* were often more prone to errors and hence comparatively less likely to be distinguished from sequencing errors. For example, in *TP53*, R248Q and R248W are among the most common mutations found in cancer patients [33]. The transition base changes that result in these mutations could be confounded by the HTS errors at an 8-fold higher rate than the transversion alterations that lead to R248L, and 55-fold higher than those that lead to R248G (Fig. 4a). Similarly, the K700E mutation in *SF3B1* is the most frequently mutated residue in the gene’s exon 10 [34, 35]; it results from a T >C mutation in a TTC trinucleotide that showed the highest rate of error for a non-synonymous amino acid change in its codon (4.74 ± 0.42×10^−5^ [error/base]). In contrast, the comparatively rarer I704F mutation – a T >A in a ATG reference trinucleotide – had one of the lowest rates of error in its respective codon (5.15 ± 1.13×10^−6^ [error/base]; Fig. 4b). K700E’s 9-fold higher rate of error than that of I704F indicated marked reduction in its relative detection sensitivity.

### Optimal sequencing depth

Insufficient sequencing depth reduces the sensitivity of detecting variants and leads to loss of statistical significance for a confident variant calling [36]. Consequently, sequencing at higher depths is expected to provide robust error rate estimates and improved sensitivities in detecting true mutations. Accurate estimation of optimal sequencing depth, beyond which the inferred background error is not further reduced, not only provides a precise view of intrinsic limitations in HTS assays, but also leads to preserving time and resources by avoiding unproductive ultra-deep sequencing experiments.

**Fig. 5:**
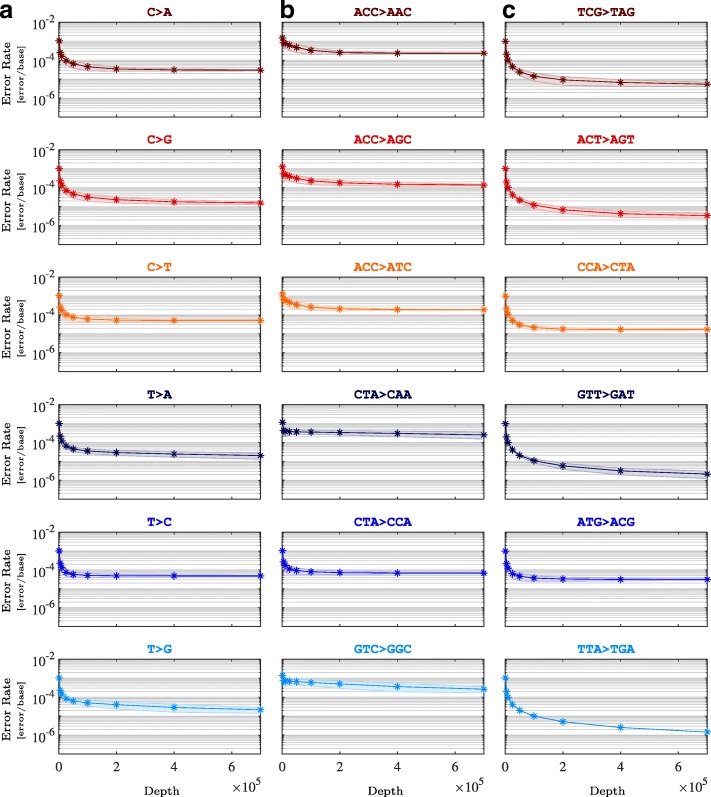
Context-specific optimal sequencing depth. Substitution error rates are classified based on their type (column **a**) and context (columns **b** and **c**) at nine different depths: 1,000 ×, 5,000 ×, 10,000 ×, 25,0000 ×, 50,000 ×, 100,000 ×, 200,000 ×, 400,000 ×, and 700,000 ×. *In silico* depth reduction procedure was performed on merged reads, amplified by polymerase Ultra II to an average depth of 1,930,473 ×. The shaded areas are uncertainty bounds of one standard deviation around the average, derived from 500 independent sub-samples

To provide insight on optimal sequencing depth, we performed *in silico* experiments and estimated context-specific error rates as a function of depth. We randomly selected merged reads and constructed simulated sequencing data at depths ranging from 1,000 × to 700,000 ×, with 500 independent replicates at each depth to establish confidence intervals (Fig. 5). MERIT showed that the type of substitution error was an important determinant in estimating the optimal depth (Fig. 5a). The error rate estimates for all transitions as well as C >A transversions did not significantly change as sequencing depth increased beyond 200,000 ×; however, the inferred rates for the remaining transversions marginally improved at higher depths. More importantly, this analysis highlighted the importance of context-specific error profiling in determining detection sensitivity thresholds for true mutations. For example, at 5000 ×, the corresponding error rates for all T >A errors, T >A errors in CTAs, and T >A errors in GTTs were 2.19 ± 0.37×10^−4^ [error/base], 4.27 ± 2.28×10^−4^ [error/base], and 1.96 ± 0.02×10^−4^ [error/base], while at 700,000 ×, these rates were reduced to 2.02 ± 0.73×10^−5^ [error/base], 2.5 ± 0.99×10^−4^ [error/base], and 2.1 ± 0.89×10^−6^ [error/base], respectively. Selecting a frequency threshold for these variants at 5000 × based on the general T >A rate may not yield significant number of false calls independent of their sequence contexts; however, at depths > 5000×, setting a threshold based on all T >A errors would lead to substantial false positive CTA >CAA and false negative GTT >GAT calls, as their corresponding error rates diverge at high depths, reaching a difference of two orders of magnitude at 700,000 ×.

It should be noted, however, that SAMtools might not be able to detect all indels at all depths [21, 22]. Although comparing indel error rates might be only statistically meaningful at fixed sequencing depths, we did observe a reduction in estimated rate of error for single-nucleotide deletions relative to sequencing depth (Additional file 1: Figure S6). Calling all complex indels, especially when they are present in only a few reads, may require more sophisticated variant callers whose results can be combined with substitution calls to obtain a comprehensive error profile by MERIT.

### DNA polymerase fidelity estimation

High-fidelity DNA polymerases – equipped with proofreading – result in fewer base misincorporations in PCR enrichment step, and thus, can reduce HTS error rates. The Hi-Fi 2X, Ultra II, KAPA, and SuperFi enzymes are marketed as high-fidelity polymerases, specifically designed for efficient amplification of complex templates such as those with GC-rich regions. Their providers have reported a fidelity 100 × better than wild-type Taq DNA polymerase [37–40].

We applied MERIT to merged reads at equal depths of 650,000 ×, ensuring that the estimated fidelities were not affected by sequencing depth. When all errors were included in the analysis, global error rates suggested that these polymerases performed fairly similarly to each other, with the highest and lowest error rates belonging to KAPA and SuperFi enzymes, respectively. Specifically, the global substitution error rates for Hi-Fi 2X, Ultra II, KAPA, and SuperFi were estimated at 2.66 ± 0.21×10^−6^, 1.91 ± 0.19×10^−6^, 6.95 ± 0.54×10^−6^, and 1.76 ± 0.25×10^−6^ [error/base/doubling], respectively (Additional file 1: Figure S1a).

Because different assays, quantification methods, and descriptive units [21, 41] are often used to estimate the polymerase fidelity, comparing the reported rates in the literature is a challenging task and beyond the scope of this work. More importantly, error rate profiles in HTS data are reported to be blackplatform as well as batch dependent [42]. For example, using single cell sequencing technique error rates of 5.3 × 10^−7^ [sub/base/doubling] and 1.6 × 10^−5^ [sub/base/doubling] are reported in [21] for Ultra II and KAPA polymerases, respectively. In another study [43], a barcoding sequencing approach yielded a rate of 4 × 10^−6^ [substitutions/base] for Ultra II while 2.8 × 10^−7^ [substitutions/base] is reported for KAPA enzyme in [39]. Here, we use MERIT to emphasize on the importance of context-specific polymerase fidelity estimation and provide a robust comparison of these commonly used high-fidelity enzymes performed on a single sequencing platform.

Relying solely on global error rates for comparing the replication accuracy of these high-fidelity enzymes may be misleading [44]. Previous HTS-based analyses of polymerase fidelity estimation have classified substitutions into transition and transversion types and have showed preferential rates of error [21, 41, 44, 45]. Additional file 1: Figure S1b represents such classification of the substitution errors in our ultra-deep data, providing a more detailed understanding of the replication fidelity of these enzymes. For example, the global substitution fidelity of SuperFi was found 3.95 ± 0.65× better than that of KAPA’s; however, specific substitution fidelity differed widely. C >G errors of SuperFi were 6.88 ± 2.16× less frequent than those of KAPA. In contrast, for C >A substitutions, SuperFi’s advantage over KAPA was reduced to only 1.85 ± 0.30×.

**Fig. 6:**
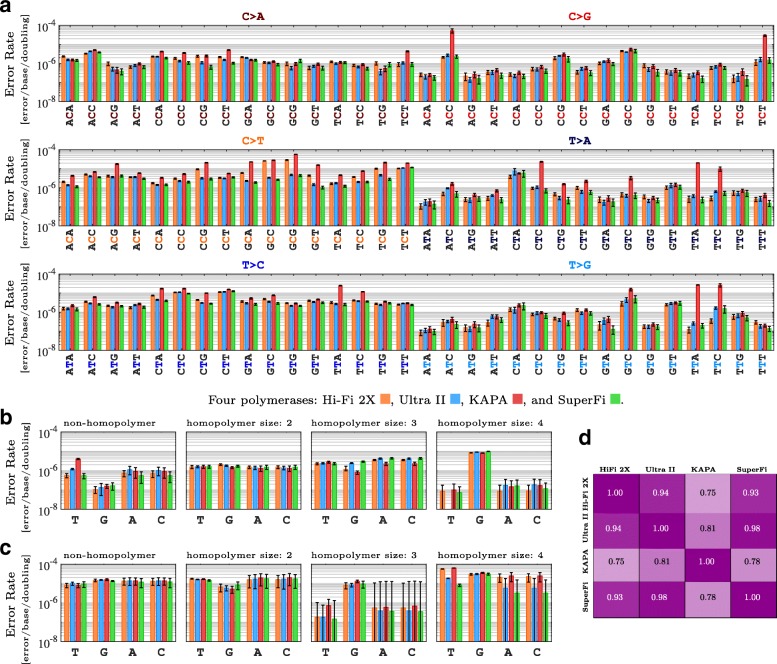
In-depth comparison of the error rates for four high-fidelity polymerases. **a**) Context-specific substitutions. **b**) Single-base insertions. **c**) Single-base deletions. **d**) Spearman’s rank correlation coefficient between context-specific error profiles. Results are obtained by averaging over 100 independent samples to establish error bars, which indicate one standard deviation from the average

**Fig. 7:**
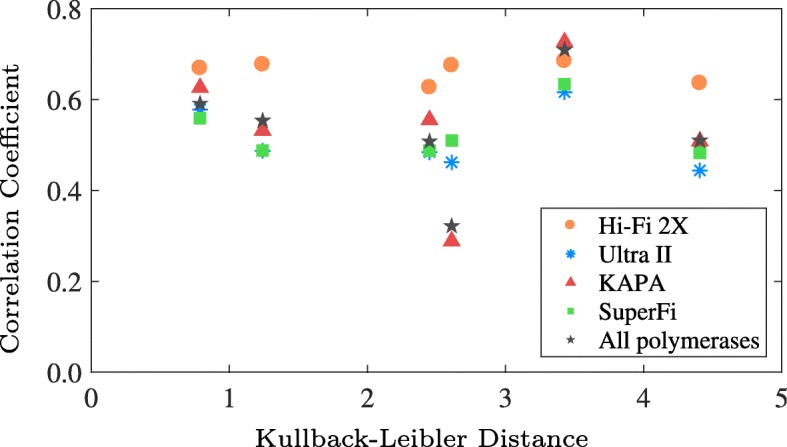
Relationship between amplicon genomic content and error profiles. Spearman’s rank correlation coefficient between the context-specific error profiles of the targeted genes as a function of the symmetric Kullback-Leibler distance between their content profiles presented in Additional file 1: Figure S2

For a more comprehensive analysis, we used MERIT to estimate 96 context specific substitutions and observed substantial variations (Fig. 6a). For example, TTA >TGA error rate of SuperFi was found 132 ± 35× lower than KAPA, while for GCG >GAG errors, KAPA performed just slightly better than SuperFi. Such classification of substitution errors based on their genomic context enabled us to perform robust statistical comparisons between the replication accuracy of different DNA polymerases using Spearman’s rank correlation coefficients presented in Fig. 6d, rather than just comparing them using a single global error rate. Moreover, using the data from multiple regions of the *TP53* and *SF3B1* genes, we found limited change in overall error profiles as the similarities between the genomic content of the amplified amplicons decreased (Fig. 7).

### Application of MERIT to clinical samples

Sample preservation and library preparation of clinical samples can lead to specimen-specific errors. MERIT provides a tool to assess and compare such error profiles. Additional file 1: Figure S5 represents the substitution error rates estimated for hematopoietic samples collected from leukemia patients presented in [25]. The error rate estimates for these clinical samples showed a high rate of transition errors similar to previous results from a cell line. A major difference, however, was that the C >A errors proceeded by C and T bases were more frequent than those proceeded by A and G. Our data did not show a preferred 3 ^′^ base trailing the misread C base. This high rate of C >A errors has been observed in previous studies [32, 46], specifically an abnormally high rate of CCG >CAG errors in both tumor and normal samples from cancer patients [32].

## Conclusions

Novel library preparation methods have succeeded in reducing the background sequencing noise, which has led to improving the sensitivity of detecting true mutations in heterogenous samples. PCR-free library preparation methods [47, 48] have forgone the bias associated with the polymerase base incorporation [49, 50], however, the large amount of input DNA required in these techniques is the main burden for their application in clinical cancer genomic testing. As the exponential PCR amplification is a crucial step in HTS, other techniques have focused on minimizing polymerase errors rather than abolishing the PCR step entirely, including Safe-Seq [6], Duplex-Seq [51], Circle-Seq [52], Cypher-Seq [53], and maximum-depth sequencing [54]. Despite all improvements, the background noise is not completely eliminated. The additional cost and complexity of these methods as well as their lower yield [54] limit their utilization in clinical cancer genomic testing. Specifically, a limited starting material, as is usually the case for tumor specimens, could results in poor sample representation due to inefficiencies in adapter ligation and loss of genetically diverse small clones [55].

In this paper, we provided a comprehensive method for profiling sequencing artifacts and discussed their impact on accurate variant detection in amplicon-based HTS data. We proposed an approach for determining the optimal sequencing depth, where errors occur at rates similar to those of true mutations. Our data obtained from Illumina platforms confirmed previous results on the differential rates of errors in paired-end sequencing reads [11], and indicated that merging the overlapping read pairs, independent of alignment approach, can notably correct errors that accumulate in sequencing instruments [55].

We also reported the application of MERIT to ultra-deep sequencing data obtained from the amplification of multiple clinically relevant loci using four high-fidelity polymerase enzymes. Although there is limited variation in both the rates of error and dependence on the genomic content of the amplified region, our results indicated that profiling polymerase misincorporation pattern according to genomic context has important clinical consequences. Specifically, we showed that error rates obtained from deep-sequencing of clinical specimens may reflect processes that affect DNA quality during sample preparations.

Sample heterogeneity, especially when low-abundance mutations are present, can confound MERIT’s sequencing error profiles. Therefore, when MERIT is applied to clinical specimens from which true mutations are not removed, the estimated rates represent the upper bound of true sequencing error rates. Our results also demonstrated that assigning a single allele frequency threshold to detect mutations may result in substantial false positive as well as false negative calls. Not only were neighboring mutational hotspots in one gene affected with markedly different error rates, there was significant variation in the sensitivity of detecting common amino acid changes within each residue. These data suggested that some of these mutations may in fact be more prevalent at sub-clonal levels in disease populations than previously reported. For instance, small mutated clones in the *TP53* gene, present in > 0.1% of alleles, are shown to be strong predictors of poor survival and possible resistance to therapy in various neoplasms [56–59]; thus, their detection at very low abundances is pertinent for patient care. Put together, our results strongly advocated mutation-specific approaches that go beyond estimating fixed detection thresholds for all variants [60–62].

As deep sequencing of patient samples becomes a routine part of precision medicine in the clinic, we believe that the application of our data-driven pipeline to tumors increases the speed with which patient data can be evaluated for presence of small prognostic mutations, hence, contributing significantly to combating drug resistance and increasing positive outcomes.

## Additional file

Additional file 1 SI Materials. (PDF 4187 kb)

## Abbreviations

BWA: Burrows-wheeler aligner
GATK: Genome analysis toolkit
Hi-Fi 2X: NEBNext® High-Fidelity 2X PCR master mix polymerase
HTS: High-throughput sequencing
KAPA: KAPA HiFi PCR kits with ReadyMix polymerase
MERIT: Mutation error rate inference toolkit
PCR: Polymerase chain reaction
SuperFi: Invitrogen™Platinum™ SuperFi™ DNA polymerase
Ultra II: NEBNext® Ultra™ II Q5® master mix polymerase
VAF: Variant allele frequency
