# Supplementary material for: MERIT reveals the impact of genomic context on sequencing error rate in ultra-deep applications
Source: BMC Bioinformatics. 2018 Jun 8;19:219. doi: 10.1186/s12859-018-2223-1 (PMC5994075; doi:10.1186/s12859-018-2223-1)
Supplement: Supplementary file 1 — SI Materials. (PDF 4187 kb) [file 12859_2018_2223_MOESM1_ESM.pdf]

SI Materials

MERIT reveals the impact of genomic context on sequencing error rate in ultra-deep applications

Mohammad Hadigol[1] and Hossein Khiabanian[1,2]

[1]Center for Systems and Computational Biology, Rutgers Cancer Institute of New Jersey, Rutgers University, New Brunswick, NJ

[2]Department of Pathology and Laboratory Medicine, Rutgers Robert Wood Johnson Medical School, Rutgers University, New Brunswick, NJ

Remark 1 *Sum of Binomial Random Variables:* Let $X_1, X_2, \cdots, X_m$ be independent binomial random variables where X_i has a Binomial(n_i, p) distribution for $i = 1, 2, ..., m$. Then $X_1 + X_2 + \cdots + X_m$ has a Binomial($\sum_{i=1}^{m} n_i$, p) distribution.

Proof 1 Assuming $m = 2$, from the discrete convolution formula, one can write the distribution for the random variable $\Xi = X_1 + X_2$ as

$$P(\Xi = \xi) = f_\Xi(\xi) = \sum_{x_1=0}^{\xi} f_{X_1}(x_1) f_{X_2}(\xi - x_1),$$

$$f_\Xi(\xi) = \sum_{x_1=0}^{z} \binom{n_1}{x_1} p^{x_1}(1-p)^{n_1-x_1} \binom{n_2}{\xi - x_1} p^{\xi - x_1}(1-p)^{n_2-(\xi-x_1)},$$

$$f_\Xi(\xi) = p^{\xi}(1-p)^{n_1+n_2-\xi} \sum_{x_1=0}^{\xi} \binom{n_1}{x_1}\binom{n_2}{\xi - x_1}.$$

By employing the Vandermonde's identity, $f_\Xi(\xi)$ is summarized to

$$f_\Xi(\xi) = \binom{n_1+n_2}{x_1+x_2} p^{x_1+x_2}(1-p)^{n_1+n_2-x_1-x_2}, \tag{1}$$

which is a Binomial($n_1 + n_2$, p) distribution. The case for several binomial random variables, i.e., $m > 2$, follows by induction.

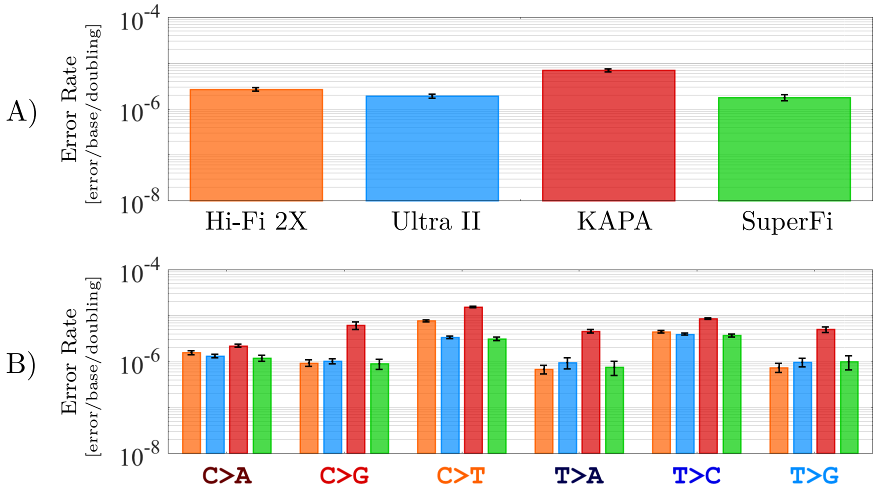

Figure S1: Estimating the fidelity of four polymerases, i.e., Hi-Fi 2X ■, Ultra II ■, KAPA ■, and SuperFi ■. A) Total substitution error rate. B) Substitution error rates classified by type. Results are obtained by averaging over 100 independent replications to establish error bars, which indicate one standard deviation from the average

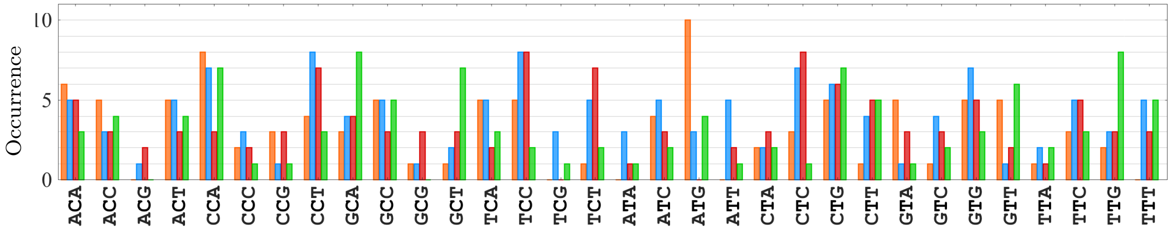

Figure S2: Number of trinucleotide reference tuples in four different amplicons extracted from reference genome hg19. TP53.1 ■, TP53.2 ■, TP53.3 ■, and SF3B1.1 ■.

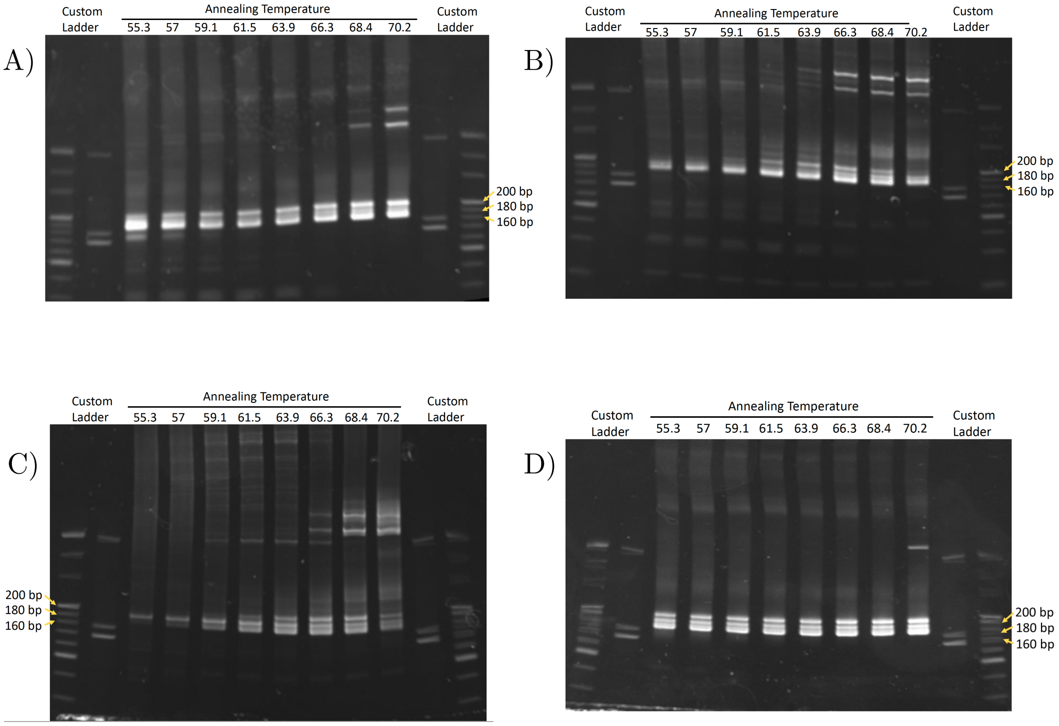

Figure S3: Gradient PCR optimization of the primer annealing temperature for: A) Hi-Fi 2X, B) Ultra II, C) KAPA, and D) SuperFi.

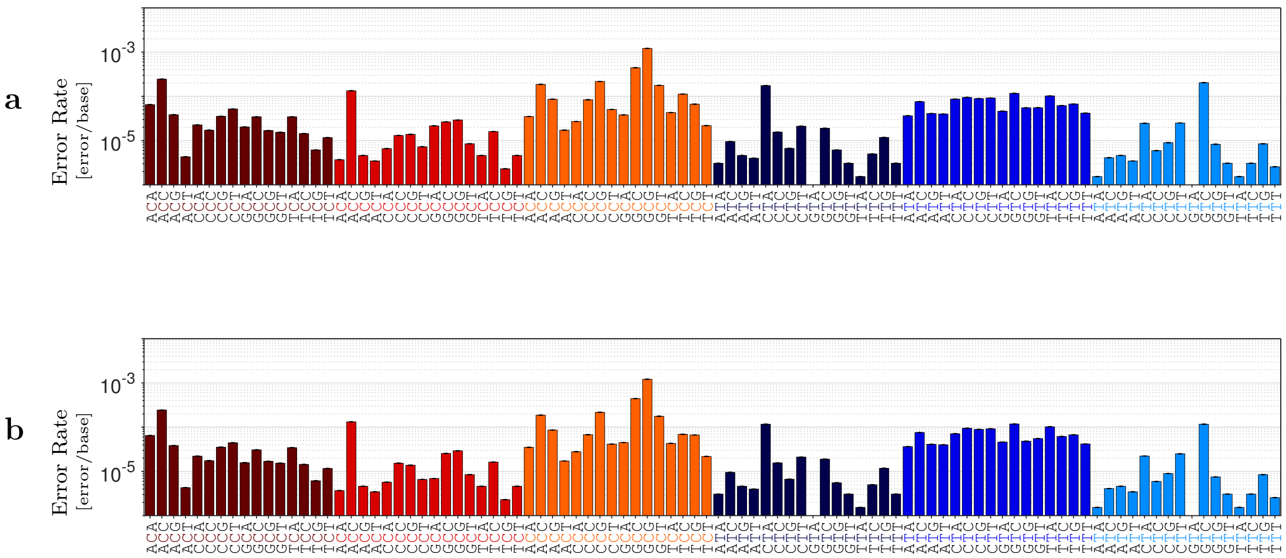

Figure S4: Estimated context-specific substitution error rates for polymerase Hi-Fi 2X. **a**) BWA aligner. **b**) Bowtie aligner. Depth of merged reads for polymerase Hi-Fi 2X were reduced *in silico* to approximately 650,000×

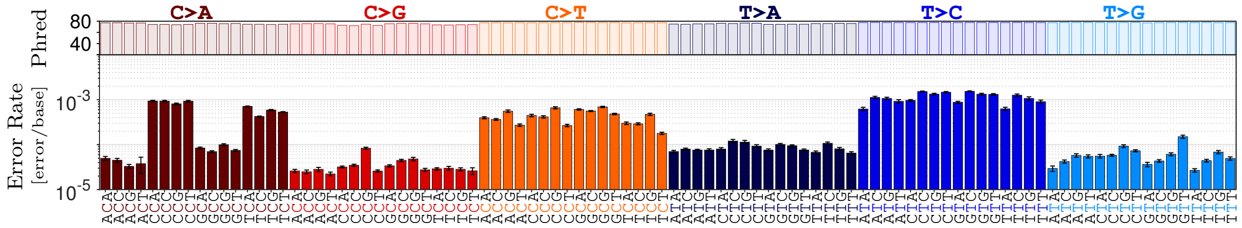

Figure S5: Estimated context-specific substitution error rates for 29 hematopoietic samples collected from 9 patients with chronic lymphocytic leukemia. Error rates are estimated after merging of overlapped reads. Results are obtained by averaging over all samples to establish error bars which indicate one standard deviation from the average.

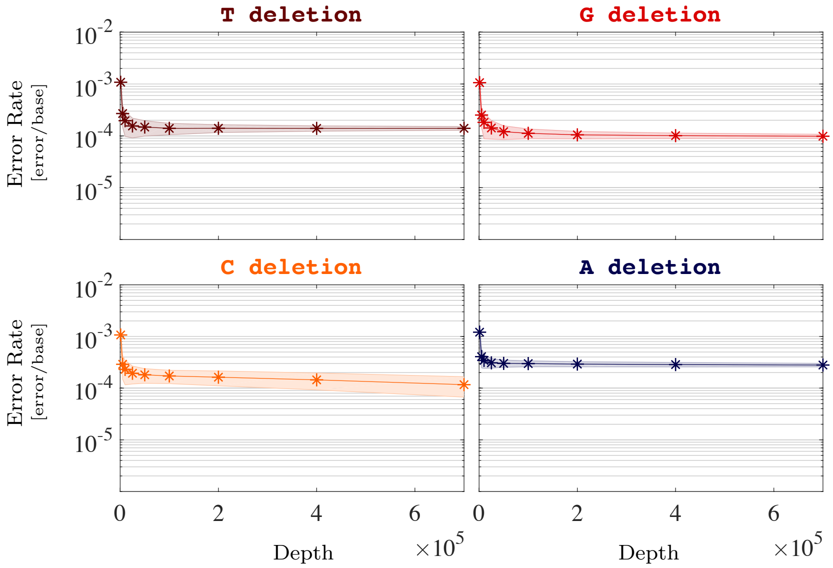

Figure S6: Single-base deletion error rates are classified based on their type at nine different depths: 1,000×, 5,000×, 10,000×, 25,0000×, 50,000×, 100,000×, 200,000×, 400,000×, and 700,000×. *In silico* depth reduction experiment was performed on merged reads, amplified by polymerase Ultra II to an average depth of 1,930,473×. The shaded areas are uncertainty bounds of one standard deviation around the average, derived from 500 independent sub-samples.

Table S1: Four targeted loci in *TP53* and *SF3B1* genes considered in this study.

Gene	Targeted position	Forward primer (GC%)	Reverse primer (GC%)	Product size (bp)
TP53.1	chr17:7577505-7577610	TCTTCCAGTGTGATGATGGTG (48%)	AGGTTGGCTCTGACTGTACCA (52%)	106
TP53.2	chr17:7578190-7578326	TAGGGCACCACCACACTATG (55%)	GTCCCCAGGCCTCTGATT (61%)	137
TP53.3	chr17:7577069-7577187	CGGAGATTCTCTTCCTCTGTG (52%)	GCCTCTTGCTTCTCTTTTCC (50%)	119
SF3B1.1	chr2:198266780-198266890	GAGTTGCTGCTTCAGCCAAG (61%)	TTGGGGCATAGTTAAAACCTG (43%)	111

Table S2: Average coverage in merged and PE reads for different loci.

	DNA Polymerase	TP53.1	TP53.2	TP53.3	SF3B1.1
**Merged reads**	Hi-Fi 2X	5,630,816	5,545,560	1,240,271	3,098,563
	Ultra II	1,978,382	1,930,473	667,125	1,646,262
	KAPA	823,185	11,625,981	929,422	2,718,782
	SuperFi	5,695,236	5,396,628	2,401,502	6,827,542
**Paired-end reads**	Hi-Fi 2X	11,260,867	11,089,852	2,480,376	6,196,521
	Ultra II	3,956,452	3,860,571	1,333,990	3,292,191
	KAPA	1,646,223	23,249,090	1,848,263	5,437,078
	SuperFi	11,389,742	10,792,208	4,802,398	13,653,800

Table S3: Optimization of the first round PCR cycle for different polymerases used in this study.

Polymerase	PCR cycle	Concentration (ng/μL)	Volume (μL)	Total amount (ng)
Hi-Fi 2X	16	0.206	14	2.884
	20	0.571	14	7.994
	24	4.78	14	66.92
	28	33.9	14	474.6
Ultra II	16	0.388	14	5.432
	20	1.91	14	26.74
	24	14.8	14	207.2
	28	53	14	742
KAPA	16	0.235	14	3.29
	20	0.562	14	7.868
	24	2.9	14	40.6
	28	22.8	14	319.2
SuperFi	16	0.186	14	2.604
	20	0.411	14	5.754
	24	3.17	14	44.38
	28	24.6	14	344.4

Table S4: Second round of PCR for multiplexing includes seven cycles for all four polymerases. Replication efficiency is the ratio of template doubling (d) over the number of PCR cycles performed. To achieve a uniform coverage of the considered regions for all polymerases, we started with a similar amount (about 5.5 ng) for each polymerse for the second round of PCR.

**Poly-merase**	**PCR cycle**	**Starting DNA amount [ng]**	**Final product concentration [ng/μL]**	**Volume [μL]**	**Final product amount [ng]**	**Repli-cation efficiency**
Hi-Fi 2X	7	5.5387	11.70	20	234.0	0.771
Ultra II	7	5.4320	8.27	20	165.4	0.704
KAPA	7	5.4514	5.66	20	113.2	0.625
SuperFi	7	5.7540	12.50	20	250.0	0.777

Table S5: Parameters of SAMtools mpileup used in MERIT pipeline compared with their default values.

Input option	Description	Default SAMtools mpileup	Default MERIT SAMtools mpileup
`-A`	Do not skip anomalous read pairs in variant calling	Disabled	Enabled
`-B`	Disable probabilistic realignment for the computation of base alignment quality (BAQ)	Disabled	Enabled
`-d`	Maximum depth considered at each locus	8,000	1,000,000
`-Q`	Minimum base quality for a base to be considered	13	0
`-x`	Disable read-pair overlap detection	Disabled	Enabled
`-L`	Maximum depth for indel calling	250	1,000,000
`-F`	Minimum fraction of gapped reads	0.002	0.000001
`-o`	Phred-scaled gap open sequencing error probability	40	40
`-e`	Phred-scaled gap extension sequencing error probability	20	20
`-h`	Coefficient for modeling homopolymer errors	20	20
